# 薄壁空洞性肺癌：24例病例分析及文献回顾

**DOI:** 10.3779/j.issn.1009-3419.2014.07.10

**Published:** 2014-07-20

**Authors:** 俊唐 郭, 朝阳 梁, 向阳 初, 乃康 周, 玉鹗 孙, 阳 刘

**Affiliations:** 100853 北京，解放军总医院胸外科 Department of Thoracic Surgery, Chinese PLA (People's Liberation Army) General Hospital, Beijing 100853, China

**Keywords:** 肺肿瘤, 空洞, 囊性病变, 计算机断层扫描, Lung neoplasms, Cystic, Cysts, Computed tomography

## Abstract

**背景与目的:**

英国学者Anderson和Pierce于1954年首先报道了表现为囊性病变的肺癌。作为少见的肺癌特殊表现类型，薄壁囊性肺癌在临床中经常被误诊。本研究旨在探讨薄壁空洞性肺癌的临床特点、影像学特点，诊断及治疗。

**方法:**

自2007年3月-2013年12月，解放军总医院胸外科共收治4, 897例原发性肺癌患者，其中24例影像学表现为薄壁空洞型肺癌。我们将影像学上表现为囊性且75%以上囊壁厚度小于4 mm的肺癌定义为薄壁空洞性肺癌。回顾性总结24例病例的临床资料、病理结果及随访情况。

**结果:**

薄壁空洞型肺癌在我科同期接受手术的肺癌中比例为0.49%（24/4, 897）。其中男性19例，女性5例，平均年龄56.5岁。14例无临床症状，于查体时发现。10例有呼吸道症状。24例患者中18例为腺癌（包括原位腺癌及微浸润腺癌），3例鳞癌，1例大细胞癌，1例小细胞癌，1例腺鳞癌。平均随访时间28个月，3例因肺癌死亡，其余21例未见复发。

**结论:**

薄壁空洞型肺癌是肺癌少见的影像学表现类型，临床中应避免误诊。其具体形成机制并不明确，可能是肺癌形成的某一阶段。薄壁空洞型肺癌如能早期诊断，可获得较好预后。

## Abstract (en)

**Background and objective:**

Lung cancer presenting as cystic lesions was first described by Anderson and Pierce in 1954. Lung cancer presenting as cysts is a rare entity in clinical practice. Differential diagnosis is difficult in the benign-like cyst. This study investigated the clinical characteristics, diagnosis and treatment of lung cancer presenting as cysts.

**Methods:**

We conducted a retrospective analysis of the clinical records of 24 patients who underwent surgery for a primary lung cancer presenting as cysts in our department between 2007 and 2013. We defined a 'Thin-walled cyst' as a cavitary lesion with a wall thickness of 4 mm or less along at least 75% of the circumference of the lesion. The whole group underwent post-operative follow-up.

**Results:**

The incidence of cystic lung cancer was 0.49% (24/4, 897) of surgical cases. The subjects' age ranged from 19 to 77 yr with a median age of 56.5 yr. Ten cases presented with respiratory symptoms while 14 showed abnormal shadows on a chest CT without symptoms. Histological analysis showed that 18 cases were of adenocarcinoma, three of squamous cell carcinoma, one of small cell carcinoma, one of adenosquamous carcinoma and one of large cell carcinoma. Three patients were dead, and the remaining 21 patients are alive and disease free at the end of follow-up.

**Conclusion:**

Cystic lung cancer should be kept in mind during the differential diagnosis of focal benign cyst. Cystic lung cancer could achieve a good outcome if early diagnose can be obtained.

肺囊性病变在肺计算机断层扫描（computed tomography, CT）上常见，包含了多种疾病。一般认为壁厚度＜4 mm的含气腔病变为囊性病变，而厚度＞4 mm或者周围有浸润或肿物的含气腔称为空洞。肺孤立性囊性病变一般认为是良性病变^[1, 2]^。1954年，英国的Anderson和Pierce^[3]^首先报道了表现为囊性病变的肺癌。作为少见的肺癌特殊表现类型，薄壁囊性肺癌在临床中经常被误诊。我们总结了过去6年连续收治的24例表现为薄壁囊性病变的肺癌，并首次将此类肺癌定义为薄壁空洞性肺癌，现将结果报道如下。

## 1 资料与方法

### 1.1 研究对象

我们将影像学上表现为囊性且75%以上囊壁厚度＜4 mm的肺癌定义为薄壁空洞性肺癌。自2007年3月-2013年12月，解放军总医院胸外科共收治4, 897例原发性肺癌患者，选取其中24例影像学表现为薄壁空洞型肺癌为研究对象。

### 1.2 方法

本文回顾性总结24例病例的临床资料、病理结果及随访情况。每位患者均行胸部X线、胸部CT，7例患者行氟代脱氧葡萄糖-正电子发射计算机断层显像（fluorodeoxyglucose positron emission tomography, FDP-PET）/CT检查。TNM（tumor-node-metastasis）分期采用第7版国际抗癌联盟（Union for International Cancer Control, UICC）肺癌分期。

## 2 结果

### 2.1 一般情况

24例病例中男性19例，女性5例，平均年龄56.5岁。薄壁空洞型肺癌在我科同期接受手术的肺癌中比例为0.49%（24/4, 897）。同期行肺孤立性空洞手术396例，薄壁空洞型肺癌占6%。14例无临床症状，于查体时发现。10例有呼吸道症状，其中4例表现为咳嗽，5例咳嗽伴痰中带血，1例有低热。6例患者有重度吸烟史。仅有1例有肺大疱病史。2例既往有肺癌病史，术后复查发现对侧肺囊性病变。

### 2.2 影像学表现

每位患者均行胸部CT及胸片检查。胸部CT表现为直径2 cm-6 cm的囊性病变。5例患者有连续的CT观察，表现为持续增大，壁有所增厚（图 1）。7例行FDG PET-CT检查，除1例囊壁外缘有高代谢外其余6例均无异常高代谢。所有24例均为周围性肺癌。其中7例位于右肺上叶，1例右肺中叶，5例右肺下叶，5例左肺上叶，6例左肺下叶。3例患者有双原发肺癌（另一原发灶均为实性）。

**1:**
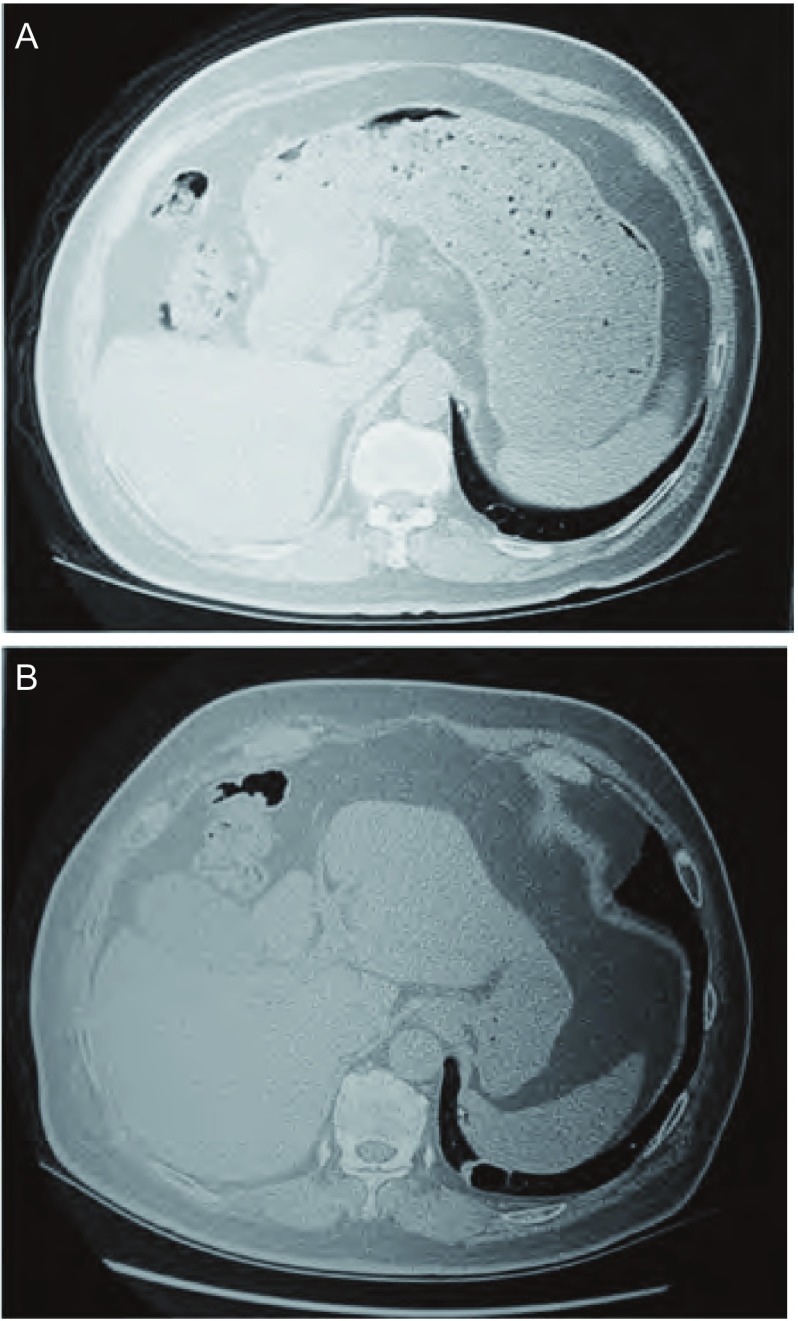
患者68岁，男性。A：2008年查体胸部CT显示左肺下叶薄壁空洞性病变；B：2011年胸部CT显示左肺下叶薄壁空洞性病变变大，内侧壁不均匀增厚。术后病理证实为高分化腺癌。 68-yr-old, male. A: Computed tomography (CT) in 2008 revealed a thin-walled cystic lesion in left lower lobe; B: CT in 2011 showed that cystic lesion in left lower lobe enlarged and wall thickened. Pathological examination proved as well differentiated adenocarcinoma.

### 2.3 诊断

术前观察期1周-5年，从发现病变至获得诊断平均为9.2周。4例被误诊为结核，2例被误诊为肺大疱，2例被误诊为真菌感染。接受抗炎治疗4例。

### 2.4 手术及病理

24例中15例行肺叶切除术，7例行楔形切除术，1例行全肺切除术，1例因胸膜转移行胸膜活检术。术后病理回报：18例为腺癌，其中包括4例原位腺癌及2例微浸润腺癌，3例鳞癌，1例大细胞癌，1例小细胞癌，1例腺鳞癌。18例无淋巴结转移，2例N1淋巴结转移，4例N2淋巴结转移。TNM分期：Ⅰa期16例，Ⅰb期1例，Ⅱa期2例，Ⅲa期3例，Ⅲb期1例，Ⅳ期1例。

### 2.5 辅助治疗及随访

7例进展期肺癌接受了辅助治疗。6例接受化疗，1例接受放化疗，1例接受表皮生长因子受体-酪氨酸激酶抑制剂（epidermal growth factor receptor-tyrosine kinase inhibitor, EGFR-TKI）治疗。平均随访时间28个月，3例因肺癌死亡，其余21例未见复发。

## 3 讨论

肺孤立性囊性病变一般认为是良性病变。恶性病变较良性病变比较有较高的厚壁空洞发生率^[4]^。Woodring等^[5]^报道空洞壁最厚部分厚度可以用来鉴别良性空洞和恶性空洞。在65例患者中，空洞壁小于1 mm的均为良性，壁厚为2 mm-4 mm的空洞中14%为恶性，壁厚为5 mm-15 mm的空洞中49%为恶性，而壁厚大于15 mm的95%为恶性。本研究显示薄壁空洞性肺癌在肺孤立性空洞病变外科病例中比例为6%，属于少见疾病。其他常见的疾病包括肺大疱、先天性囊性病变、结核和肺真菌病。除了肺癌，囊性腺样癌和部分转移瘤也可以表现为囊性病变。本报道未涉及转移瘤。

1954年，英国的Anderson和Pierce首先报道了表现为囊性病变的肺癌。在过去20年里，有数个关于肺薄壁空洞性肺癌的相关文献，大多数为个案报道，且多数来自亚洲国家^[6-16]^（表 1）。日本学者Sugimoto^[12]^于2007年报道了8例影像学上表现为薄壁空洞的肺癌，为过去报道病例数最大的一组。本文收集了24例影像学表现为囊性的肺癌，并将此类肺癌命名为薄壁空洞性肺癌。

**1:** 薄壁空洞性肺癌相关文献小结 Summary of latest literatures about thin-walled cystic lung cancer

No.	Author	Year	No. of cases	Age	Sex	Histology	Therapy	Stage
1	Singh^[6]^	2012	1	45	M	SCC	Chem	cⅢb
2	Goto^[7]^	2011	1	60	M	ADC	Lob	pT2aN0M0
3	Lan^[8]^	2010	1	27	F	ADC	Chem+RT	pT2N3M0
4	Kondo^[9]^	2010	1	60	F	ADC	Chem	cⅣ
5	Matsuoka^[10]^	2010	1	79	F	ADC	Lob	pT1N0M0
6	Iwata^[11]^	2009	1	68	M	SCC	Lob	pT2N2M0
7	Sugimoto^[12]^	2007	8					
				45 62 84 78 78 81 72 79	M M M M F M M M	ADC ADC ADC ADC ADC SCC SCC ASC	Chem Lob WR WR Lob Lob Lob Lob	cT4N3M1 pT2N2M1 cT3N2M0 cT3N2M0 pT1N0M0 pT1N0M0 pT1N0M0 pT2N0M0
8	Tanaka^[13]^	2006	1	71	M	LC	Lob	pT1N0M0
9	Jakopovic^[14]^	2005	1	40	F	LC	Lob	pT2N0M0
10	Kobashi^[15]^	2005	1			ASC	Lob	pT2N2M0
11	Prichard^[16]^	1984	2					
				30	F	BAC BAC	Lob; pneumonectomy	Ⅰb Ⅱb
ADC: adenocarcinoma; SCC: squamous cell carcinoma; LC: large cell carcinoma; ASC: adenoaquamous cell carcinoma; BAC:bronchoalveolar carcinoma; Lob: lobectomy; WR: wedge resection; Chem: chemotherapy; RT: radiotherapy; M: male; F: female.								

与实性肺癌一样，薄壁空洞性肺癌也可以表现为呼吸道症状。部分患者无症状而偶尔查体时发现。本组中发现至获得诊断平均为9.2周。确诊前常应用抗炎、抗结核等治疗。这表明薄壁空洞性肺癌在影像学上属于不典型肺癌而诊断困难。因此，临床工作中应注意此类肺癌。

虽然恶性囊性病变与良性病变鉴别困难，高分辨CT仍是薄壁空洞性肺癌诊断主要手段。当遇到无肺气肿及肺大疱病史患者新发肺囊性病变时，我们应怀疑恶性。此类患者应密切随访。如果肺囊性病变显示出一些恶性征象，如壁不规则增厚、出现结节、增大等，有必要手术干预。PET-CT在薄壁空洞性肺癌诊断中阳性率较低，可能与病例数少有关。普通经皮肺穿刺活检因取材困难，对薄壁空洞性肺癌诊断效率低，且有可能造成气胸。Nakahara等^[17]^应用CT引导经皮细针盥洗技术，对薄壁空洞性病变诊断准确率明显升高。

传统观念认为肺鳞状细胞癌易形成薄壁空洞^[18]^。Anderson和Pierce报道的5例病例中组织学类型全部为鳞状细胞癌。然而新近报道的薄壁空洞性肺癌中腺癌比例明显增加，尤其是细支气管肺泡癌（目前新命名为原位腺癌）。本组中腺癌占绝大多数。各种病理类型的肺癌均可表现为孤立的薄壁空洞。

薄壁空洞性肺癌是一种影像学上特殊的肺癌类型，也可能是肺癌发生发展的特殊阶段。其形成的机制不明，可能的解释有^[6, 11, 16, 18, 19]^：①气道狭窄后止回阀机制；②实性病变由于坏死、脓肿形成、酶消化等原因而中心分解；③肺周围组织的弹性回缩引起空洞牵拉，壁变薄；④已有的囊性结构如支气管囊肿或肺大疱发生癌变。止回阀机制被大多数学者所接受^[6, 11, 20]^。我们数据中缺少支持该机制的病理学证据。

不像厚壁空洞性肺癌，本组病例有相对较好的预后。主要原因是两种类型的肺癌的形成机制不同。厚壁空洞性肺癌血管浸润更常见^[18]^。而大多数薄壁空洞性肺癌是由阀门效应引起。其次，本组病例TNM分期较早，24例中16例为Ⅰa期肺癌。

薄壁空洞性肺癌是一种少见的肺癌类型。在肺囊性病变鉴别诊断时应考虑到这种不典型肺癌。如果能早期诊断，薄壁空洞性肺癌能获得较好预后。
